# Association of Visceral Adiposity Index and Handgrip Strength with Cardiometabolic Multimorbidity among Middle-Aged and Older Adults: Findings from Charls 2011–2020

**DOI:** 10.3390/nu16142277

**Published:** 2024-07-15

**Authors:** Junping Liu, Wei Liu, Lu Wang, Nan Wang, Lin Wu, Xinru Liu, Zhaoyue Liu, Yue Zhou, Xinle Yin, Yaping Liu, Qunhong Wu, Yu Cui, Libo Liang

**Affiliations:** Department of Social Medicine, School of Health Management, Harbin Medical University, No. 157 Baojian Road, Nangang District, Harbin 150081, China; ljp0260827@163.com (J.L.); 15764504915@163.com (W.L.); 18845766973@163.com (L.W.); w820785718@163.com (N.W.); 15542164338@163.com (L.W.); liuxinru27@163.com (X.L.); m17862819670@163.com (Z.L.); 18710872183@163.com (Y.Z.); yinxinle2022@163.com (X.Y.); 15613537505@163.com (Y.L.); wuqunhong@163.com (Q.W.); cuiyu4640@163.com (Y.C.)

**Keywords:** visceral adiposity index, handgrip strength, cardiometabolic multimorbidity

## Abstract

The visceral adiposity index (VAI) and handgrip strength (HGS) are identified as important objectives for the prevention of illness. Nevertheless, there is limited understanding regarding the impact of the VAI and HGS on cardiometabolic multimorbidity (CMM). We aimed to ascertain the impact of the VAI and HGS on CMM among middle-aged and older people. Data spanning from 2011 to 2020 were derived from the China Health and Retirement Longitudinal Study (CHARLS). In total, 7909 individuals aged 45 years and older were included. Cox proportional hazard regression was utilized to examine the correlation among the VAI, HGS, and CMM. Throughout the 10-year follow-up, we determined that both the VAI (HR = 1.330; 95%CI = 1.179–1.500) and HGS (HR = 0.745, 95%CI = 0.645–0.861) exhibited significant associations with CMM risk. Individuals exposed to both a high VAI and low HGS were found to have higher hazards of CMM (HR = 1.377, 95%CI = 1.120–1.694) in contrast to participants exposed to one or none of these conditions. The older (HR = 1.414; 95%CI = 1.053–1.899) and male (HR = 1.586; 95%CI = 1.114–2.256) groups are more likely to experience CMM risk. Our findings suggest that both the VAI and HGS have significant effects on CMM risk. Appropriate interventions focused on vulnerable groups are recommended to prevent the incidence of CMM.

## 1. Introduction

Cardiometabolic multimorbidity (CMM), marked by the coexistence of a minimum of two cardiometabolic diseases (CMDs) within an individual, has witnessed a notable rise, primarily attributed to population aging, with an estimated 4.7% of older adults developing CMM [1,2]. CMDs, such as stroke, type 2 diabetes, and coronary heart disease, have been utilized to delineate CMM in accordance with prior studies [3]. Emerging evidence indicates a correlation between CMM and various adverse health outcomes, including heightened mortality risk, activity limitation, increased likelihood of dementia, decreased life expectancy, and elevated healthcare expenditure [2,4]. Given the potential catastrophic risks associated with CMM and its deleterious complications, it is urgent to identify modifiable factors contributing to its development. Existing studies predominantly concentrate on identifying risk factors for individual CMDs, resulting in a limited understanding of risk factors predicting CMM [5,6].

Multiple investigations have demonstrated that obesity serves as a pivotal modifiable risk factor for CMM and is a reliable predictor thereof [7]. One anthropometric measure that is frequently used to evaluate obesity is the body mass index (BMI). Additionally, measures like waist circumference (WC) and the body roundness index (BRI) are also employed [8]. An investigation of 101,973 adults from China has demonstrated that adiposity indicators like BMI, WC, and waist-to-height ratio (WHtR) were all linked to CMM [9]. Additionally, other findings also revealed that a high BMI and WC were also correlated with higher risks of CMM [10]. While BMI, WC, and WHtR are generally utilized as signals of obesity, they do not offer a thorough evaluation of adipose tissue spread. The VAI, a robust indication of visceral adipose tissue dysregulation, has garnered significant attention in recent years. Previous studies have investigated the direct connection among the VAI and coronary heart disease, stroke, and diabetes risk [11]. Nevertheless, the connection between VAI and CMM events still lacks clarity.

It is commonly accepted that handgrip strength (HGS) is a valid measure of both total physical fitness and muscle strength. Weak grip strength may be associated with multifarious adverse health results [12]. The risk of death, cognitive impairment, and new-onset cardiometabolic diseases strongly increase along with the decline in HGS in midlife [13]. Multiple studies have suggested that low HGS is one of the risk indicators for diabetes, heart disease, and stroke progression [14]. Based upon the UK Biobank, a cohort investigation also declared that HGS may act as a modifiable predictive element for CMM [15]. All of these studies have confirmed the adverse effects of the VAI on CMM incidence.

Obesity can elevate the risk of CMM, while weak grip strength represents a significant element for individual CMDs like stroke, heart disease, and diabetes. Nevertheless, the impact of VAI-evaluated obesity and low HGS on the development of CMM is still unknown. To solve these limitations, we took advantage of the large-scale longitudinal data to explore the relationship between VAI and HGS with CMM, and further examined their interaction for predicting CMM risk.

## 2. Methods

### 2.1. Study Design

This cohort research adopted datasets from CHARLS, a prospective nationwide cohort investigation that included residents from 28 Chinese provinces who were at least 45 years old [16]. The probability-proportional-to-size (PPS) sampling approach was implemented in the 2011 CHARLS national baseline research. A total of 17,708 people were enlisted throughout China, and since then, each of the participants has been followed up with every two to three years (wave 2 in 2013, wave 3 in 2015, wave 4 in 2018, and wave 5 in 2020). The Biomedical Ethical Review Committee of Peking University approved the ethics of the study. The main household survey’s ethical approval number was IRB00001052–11015, and the biomarker collection’s was IRB00001052–11014 [16].

**Figure 1:**
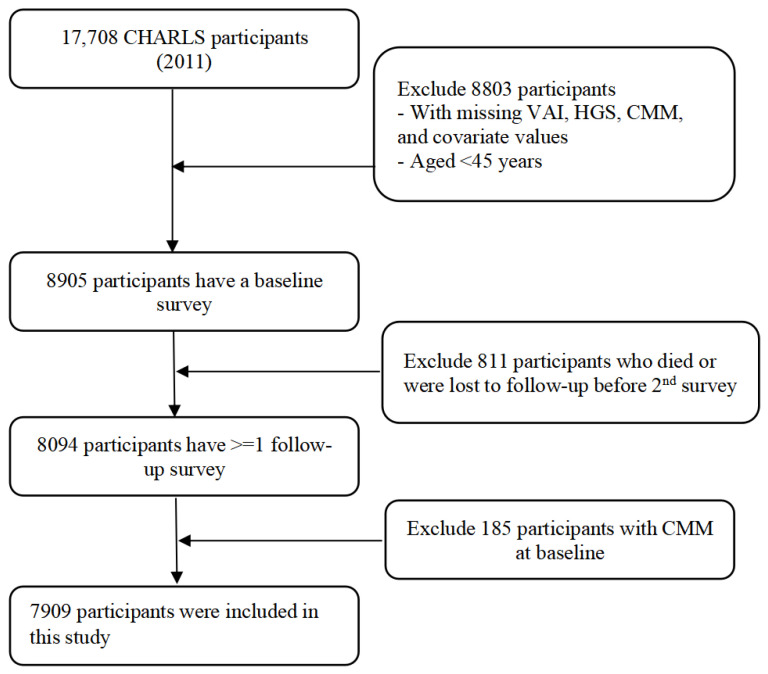
Flow diagram of respondents.

Data from the CHARLS from 2011 to 2020 was employed in the present study. Participants under 45 years old were not taken into account. Those with missing data on VAI, HGS, other variables, and CMM (the co-presence of at least two CMDs) at baseline were also excluded. Finally, 7909 participants in total were incorporated. Figure 1 illustrates the specific process of selecting respondents.

### 2.2. Calculation of Visceral Adiposity Index

The VAI, a measure of visceral adiposity [17], could completely reflect visceral fat dysfunction [18]. The VAI could be computed through the subsequent equations [19]: [WC (cm)/(39.68 + 1.88 × BMI (kg/m^2^))] × (TG (mmol/L)/1.03) × (1.31/HDL (mmol/L)) = VAI for men and [WC (cm)/(36.58 + 1.89 × BMI (kg/m^2^))] × (TG (mmol/L)/0.81) × (1.52/HDL (mmol/L)) = VAI for females. After dividing the VAI into quartiles, the participants’ VAI distribution was utilized to ascertain the cut-off point at 75% level. Those with VAI values higher than 75% (3.371) were classified as having “high VAI”.

### 2.3. Assessment of Handgrip Strength

A handheld dynamometer was applied for calculating HGS in kilos by trained volunteers [20]. Referring to the Asian Working Group for Sarcopenia 2019 consensus guidelines, testing was performed twice for every hand, while the 2 repeats’ maximum value was chosen to reflect the HGS [21]. Elbows were placed at a 90° angle on both sides while the test was conducted either standing or seated [22]. Respondents were instructed to squeeze the grips with all of their might [23]. On the basis of the 2019 guidelines proposed by the AWGS, the low-HGS cut-off marks for males and females were determined to be 28 kg and 18 kg.

### 2.4. Assessment of CMM

Similar to prior investigations [4], three queries encompassing the conditions of diabetes or high blood sugar, heart disease (such as heart attack, angina, coronary heart disease, or heart failure), or stroke diagnosed by a doctor were used to ascertain whether CMM was present. In this study, an individuals’ cardiometabolic multimorbidity status was characterized as the existence of 2 or more of the above conditions.

### 2.5. Covariates

Sociodemographic and health-related indicators were obtained at baseline via a structured questionnaire. Age, sex, residence, marital status, education, and region in China were among the sociodemographic factors. Other variables included hypertension, smoking, drinking, physical and social activities, kidney disease, hyperuricemia, falling, depression, and sleep duration, following previous studies. Chronic diseases, including hypertension, kidney disease, and hyperuricemia, were diagnosed by clinicians or determined based on the use of medications for chronic diseases.

### 2.6. Statistical Analysis

In this study, participants’ baseline characteristics were presented using frequencies and percentages. The differences across VAI groups were evaluated via chi-squared tests. Cox proportional hazard models were implemented to indicate the connection among baseline VAI and HGS and CMM risk, where hazard ratios (HRs) and 95% confidence intervals (CIs) were also assessed. Models were calculated according to the quartiles of VAI and HGS, and adjusted for sex, age, residence, marital status, education, region, smoking and drinking, physical and social activities, related chronic diseases, falling, depression, and sleep duration. Furthermore, to analyze the interaction of baseline VAI and HGS on the incidence of CMM, participants were divided into 4 categories based on the joint classification of variables: (1) neither high VAI nor low HGS, (2) high VAI only, (3) low HGS only, and (4) both high VAI and low HGS. The potential nonlinear relationship between the VAI, HGS, and CMM risk was separately estimated with a restricted cubic spline curve with three knots. To identify possible variations in the interaction effect of the VAI and HGS on CMM risk across diverse subpopulations, respondents were categorized into separate subgroups depending on sex, age, and region. Subgroup analysis was also used to test the relationship of baseline VAI, HGS with CMM at follow-up, analyzing VAI and HGS as continuous or categorical variables. To determine the robustness of the results, a sensitivity analysis was executed as well, with the VAI and HGS examined as continuous variables.

All analyses were carried out by Stata 17.0 and R Version 4.2.3. Statistical significance was determined as a *p*-value below 0.05 through two-sided testing.

## 3. Results

### 3.1. Baseline Characteristics

Table 1 presents the respondents’ baseline characteristics based on the quartiles of the VAI (Q1: ≤2.003, Q2: 2.003–3.371, Q3: 3.371–5.939, and Q4: >5.939). A total of 4205 (53.2%) of 7909 baseline respondents were females, the mean age was 59.0 years (ranging from 45 to 101), and the proportions of current smokers and drinkers were 30.6% and 33.0%, respectively. A total of 16.4% of the participants experienced falling, and 32.2% experienced depressive symptoms. Participants who were older, female, resided in rural areas, were widowed or never married, had completed college education or above, were current smokers or drinkers, had hypertension, hyperuricemia, or depression, and lived in Northeast China were more likely to have a higher VAI.

### 3.2. Longitudinal Association of Baseline VAI and HGS with CMM at Follow-Up, 2011–2020

Table 2 delineates the relationship between CMM risks and the VAI and HGS quartiles. The higher quartiles of the VAI were significantly linked to greater hazards of CMM (Q3: HR = 1.330, 95%CI = 1.179–1.500; Q4: HR = 1.204, 95%CI = 1.069–1.355) while adjusting confounding variables. Moreover, compared to Q1, Q4 displayed the strongest susceptibility to CMM risk. For continuous VAI, no significant relationship was found (Table S1). Participants with low HGS were more likely to occur CMM (Q2: HR = 0.866, 95%CI = 0.774–0.968; Q3: HR = 0.819, 95%CI = 0.724–0.926; Q4: HR = 0.745, 95%CI = 0.645–0.861), and individuals in the highest quintile of the HGS (>39 kg) had the lowest possibility of developing CMM. As far as continuous HGS is concerned, a 1 kg increase in HGS may be linked to a 1% reduce in CMM risk (Table S1). An unexpected finding was that participants who experienced depression were more vulnerable to CMM incidence (HR = 1.239, 95%CI = 1.135–1.351). Additionally, living in northeast areas was connected to a 50.5% increase in CMM risk, in contrast to those in eastern regions (HR = 1.505, 95%CI = 1.279–1.747).

A significant nonlinear dose-response relationship was identified between VAI and CMM incidence based on the findings of RCS (overall *p* < 0.05; nonlinear *p* < 0.05) (Figure S1). Moreover, the linear dose–response correlation between HGS and CMM risk was also identified (overall *p* < 0.05, nonlinear *p* > 0.05) (Figure S2).

### 3.3. The Interactions between Baseline VAI and HGS with Incidence of CMM, 2011–2020

Participants with a high VAI only (HR: 1.234, 95%CI: 1.119–1.362), low HGS only (HR: 1.376, 95%CI: 1.213–1.561), or with both a high VAI and low HGS (HR: 1.377, 95%CI: 1.120–1.694) possessed a greater chance of CMM risk than those with neither a high VAI nor low HGS after adjusting for all covariates (Table 3).

### 3.4. Sensitivity and Subgroup Analyses

**Figure 2:**
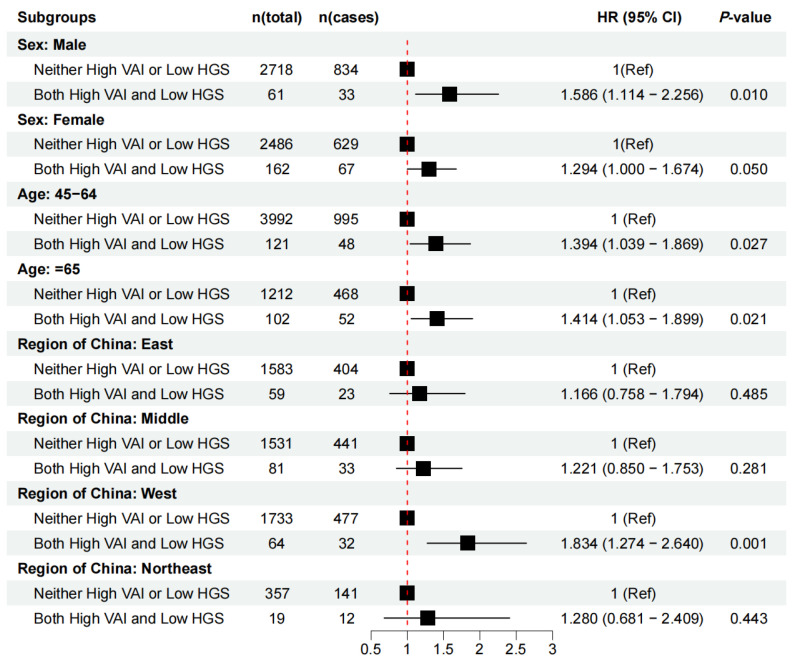
The interaction of baseline VAI and HGS on CMM by sex, age, and region.

The subgroup analysis revealed that among those 45–64 years old and those 65 and older, the relationships of both a high VAI and low HGS with CMM were stable. The associations of both a high VAI and low HGS with CMM were stronger in male populations and individuals living in western regions (Figure 2). Age, region, and sex-based stratified analyses indicated that similar associations were observed between higher VAI or lower HGS and risk for CMM, regardless of whether the VAI and HGS were investigated as continuous variables or categorized (Tables S2 and S3).

## 4. Discussion

We discovered that higher VAI scores were linked to higher risk of CMM. Lower HGS was also linked to higher CMM risk, which remained significant when considering HGS as a continuous variable. Notably, individuals living in the northeast areas and experiencing depressive symptoms tend to have higher CMM risk. Males and older individuals with both a high VAI and low HGS are more likely to develop CMM. The present study found that elevated VAI indicators have deleterious effects on the development of CMM. Previous studies also specified that higher VAI scores are linked to elevated heart disease (CHD) risk [24] and show a positive association with the incidence of type 2 diabetes mellitus [11] and stroke [25]. This could be explained by the following mechanism. First, an excessive accumulation of visceral fat may release proinflammatory cytokines and increase systemic inflammation risk, which are typical pathways for developing diabetes or cardiovascular diseases [26,27]. Furthermore, the levels of norepinephrine and epinephrine were elevated during stress tests in patients with ischemic heart failure (HF), which supports the notion of a systemic inflammatory response and its detrimental impact on cardiovascular health [28]. Additionally, excessive visceral fat may result in an increased production of adipocytokine (resistin, leptin, adiponectin), which are closely associated with heightened insulin resistance and compromised β-cell efficiency [29], leading to elevated blood glucose levels. Diabetes is closely associated with an increased risk of cardiovascular complications [30], eventually resulting in comorbidity.

Our findings also confirm the hypothesis that HGS is a predictive factor in the development of CMM. We found that diminished HGS was related to an elevated risk of CMM events. Numerous previous investigations have found that individuals with a lower HGS tend to develop cardiovascular disease [31]. In addition, an analysis of cross-sectional and observational cohort research indicates that low HGS is a susceptible factor for T2D [32]. Yanqiang Lu et al. revealed that HGS acts as a risk indicator of morbidity and all-cause mortality of CMM, using data from the UK Biobank [15]. However, it is unknown whether the same inverse correlation between HGS and CMM exists in Asian populations. Our study supplemented previous research by using data from the CHARLS and observed that a lower HGS was significantly connected to CMM risk among Asians. The underlying mechanisms for these associations could be explained by the following points. First, low HGS is an important indicator of the decline in strength and muscular mass [33]. Limited muscular strength may lead to elevated systemic inflammatory proteins and exacerbate insulin resistance [34], thereby increasing the risk of type 2 diabetes. Meanwhile, systemic inflammation may also contribute to cardiovascular diseases and related complications [35]. Secondly, decreased hand grip strength is associated with undesirable cardiometabolic markers [36], such as fasting blood glucose (FBG), glycated hemoglobin (HbA1c), and uric acid (UA). Elevated levels of FBG and HbA1c are not only basic indicators for diagnosing diabetes but also linked to stroke and coronary artery atherosclerosis [37]. Excessive UA may induce oxidative stress, vasoconstriction, and vascular wall cell proliferation [38], leading to serious diseases related to CMM [39].

We further investigated the interaction between the VAI and HGS on CMM incidence and identified that a comorbid high VAI and low HGS may lead to higher risk of CMM. The VAI, as an obesity index, is strongly related to increased risk of CMDs and cardiometabolic risk [40]. In addition, HGS is also closely linked to undesirable cardiometabolic markers [15]. Relevant research has identified that lower HGS is connected to higher stroke risk and increased all-cause mortality in individuals with CM [15]. Both a high VAI and low HGS may further exacerbate the adverse health effects of CMM among older adults.

In the subgroup analysis, we found that older adults with both a high VAI and low HGS were more vulnerable to CMM than younger individuals, primarily due to their multiple preexisting chronic conditions. Moreover, older adults tend to experience weaker muscle mass, which may be linked to serious health consequences, such as metabolic disorders. Furthermore, the associations of both a high VAI and low HGS with CMM were stronger in males. Relevant research shows that male mice are more susceptible to developing cardiac dysfunction than female mice [41]. Similar correlations between obesity and CHD with AF in men were also observed [42]. Males also tend to have a higher BMI, a larger waist circumference, and a higher proportion of smokers and drinkers compared to females, all of which contribute to their cardiovascular disease risk factors.

Notably, we found that participants with depression and residing in northeast China are more likely to experience CMM. Previous studies consistently showed that depressive symptoms are significantly associated with CMM [3], mainly due to alterations in several pathological and physiological pathways, such as oxidative stress [43]. In addition, depression may increase the risk of developing obesity and contribute to the development of CMM. Participants living in northeast China are more likely to experience lower average temperatures than those in other regions, resulting in a higher risk of CMM. Exposure to low temperatures has been widely demonstrated to be associated with excess cardiovascular risk. Cold temperatures may exacerbate high blood pressure and trigger strokes and heart failure. Furthermore, epidemiologic studies also show that higher incidences of obesity, dysglycemia, and hypertension are connected to ambient temperature, particularly in winter [44].

Several shortcomings must be taken into account. Firstly, there remain other confounders that might not have been controlled for, even though we have corrected for a variety of possible confounding factors. For example, other common comorbidities related to CMM, such as dyslipidemia and central obesity [45,46], were not included due to their overlap with VAI measurements. Additionally, during the 10-year follow-up period, changes in prevalent comorbidities such as hypertension and chronic kidney disease could have influenced the relationship between the VAI, HGS, and CMM. Secondly, variations in therapies over the follow-up period may influence the outcomes. Thirdly, the dosages of medications taken by participants may have varied over the study period. Different dosages of lipid-lowering or antidiabetic medications might have varying effects on cardiometabolic health, potentially impacting the results. Additionally, the CHARLS did not have access to medical records. Individuals were asked to disclose diseases diagnosed by a physician, potentially introducing recall bias. Lastly, the data did not encompass individuals of all age groups in China. We suggest widening the age range of study participants to improve the reliability of the results in future research.

## 5. Conclusions

We identified a significant association between high VAI and low HGS with CMM risk based on this retrospective analysis. Participants with both a high VAI and low HGS tended to have a greater risk of CMM. Simultaneously, the heightened risk associated with both a high VAI and low HGS in relation to CMM was more pronounced in males and older adults. Therefore, it is essential to implement appropriate public health countermeasures that consider the VAI and low HGS to prevent CMM incidence, especially given the rapidly aging population in China.

## Figures and Tables

**Table 1:** Characteristics of respondents by VAI quartile.

Characteristics	Total (*n* = 7909)	Q1 (*n* = 1987)	Q2 (*n* = 1999)	Q3 (*n* = 1982)	Q4 (*n* = 1941)	*p*-Value
Sex, *n* (%)						
Male	3704(46.8)	1390(37.5)	998(26.9)	739(20.0)	577(15.6)	0.000
Female	4205(53.2)	579(14.2)	1001(23.8)	1243(29.6)	1364(32.4)	
Age, *n* (%)						
45–64	5820(73.6)	1388(23.8)	1448(24.9)	1486(25.5)	1498(25.7)	0.000
≥65	2089(26.4)	599(28.7)	551(26.4)	496(23.7)	443(21.2)	
Residence, *n* (%)						
Urban	2707(34.2)	580(21.4)	628(23.2)	712(26.3)	787(29.1)	0.000
Rural	5202(65.8)	1407(27.0)	1371(26.4)	1207(24.4)	1154(22.2)	
Marital status, *n* (%)						
Married or cohabitated	7005(88.6)	1774(25.3)	1772(25.3)	1726(24.6)	1733(24.7)	0.002
Divorced or separated	86(1.1)	34(39.5)	19(22.1)	16(18.6)	17(19.8)	
Widowed or never married	818(10.3)	179(21.9)	208(25.4)	240(29.3)	191(23.3)	
Educational level, *n* (%)						
No formal schooling	2267(28.7)	457(20.2)	574(25.3)	628(27.7)	608(26.8)	0.000
Less than primary school	1508(19.1)	403(26.7)	370(24.5)	369(24.5)	366(24.3)	
Primary school completed	1782(22.5)	506(28.4)	440(24.7)	442(24.8)	394(22.1)	
Secondary/High/Vocational school completed	2273(28.7)	606(26.7)	587(25.8)	530(23.3)	550(24.2)	
College completed or above	79(1.0)	15(19.0)	28(35.4)	13(16.5)	23(29.1)	
Smoking status, *n* (%)						
Never	4791(60.6)	885(18.5)	1181(24.7)	1337(27.9)	1388(29.0)	0.000
Former	702(8.9)	225(32.1)	186(26.5)	131(18.7)	160(22.8)	
Current	2416(30.6)	877(36.3)	632(26.2)	514(21.3)	393(16.3)	
Drinking status, *n* (%)						
Never	4644(58.7)	861(18.5)	1132(24.4)	1313(28.3)	1338(28.8)	0.000
Former	654(8.3)	164(25.1)	192(29.4)	171(26.1)	127(19.4)	
Current	2611(33.0)	962(36.8)	675(25.9)	498(19.1)	476(18.2)	
Physical activities, *n* (%)	3085(39.0)	806(26.1)	760(24.6)	769(24.9)	750(24.3)	0.393
Participating in social activities, *n* (%)	3738(47.3)	849(22.7)	930(24.9)	968(25.9)	991(26.5)	0.000
Hypertension, *n* (%)	2002(25.3)	352(17.6)	425(21.2)	532(26.6)	693(34.6)	0.000
Kidney disease, *n* (%)	439(5.6)	125(28.5)	121(27.6)	103(23.5)	90(20.5)	0.086
Hyperuricemia, *n* (%)	427(5.4)	57(13.3)	75(17.6)	109(25.5)	186(43.6)	0.000
Fall(s) (last two years), *n* (%)	1297(16.4)	334(25.8)	308(23.7)	334(25.8)	321(24.7)	0.572
Depression, *n* (%)	2548(32.2)	604(23.7)	641(25.2)	690(27.1)	613(24.1)	0.023
Sleep duration (h), *n* (%)						
≤6	3978(50.3)	997(25.1)	1012(25.4)	1023(25.7)	946(23.8)	0.339
>6	3931(49.7)	990(25.2)	987(25.1)	959(24.4)	995(25.3)	
Region of China, *n* (%)						
East	2344(29.6)	591(25.2)	588(25.1)	589(25.1)	576(24.6)	0.000
Middle	2426(30.7)	547(22.5)	590(24.3)	641(26.4)	648(26.7)	
West	2570(32.5)	734(28.6)	678(26.4)	603(23.5)	555(21.6)	
Northeast	569(7.2)	115(20.2)	143(25.1)	149(26.2)	162(28.5)	

Notes: Abbreviations: Q, quartile.

**Table 2:** Relationship of baseline VAI, HGS, and incidence of CMM, 2011–2020.

Variables	HR (95%CI)	*p*-Value
VAI score		
Q1(≤2.003)	1(ref)	
Q2(2.003–3.371)	1.072(0.954–1.206)	0.244
Q3(3.371–5.939)	1.204(1.069–1.355) **	0.002
Q4(>5.939)	1.330(1.179–1.500) ***	0.000
HGS (kg)		
Q1(≤25)	1(ref)	
Q2(25–31)	0.866(0.774–0.968) *	0.011
Q3(31–39)	0.819(0.724–0.926) **	0.001
Q4(>39)	0.745(0.645–0.861) ***	0.000
Sex		
Male	1(ref)	
Female	0.709(0.620–0.812) ***	0.000
Age		
45–64	1(ref)	
≥65	1.486(1.354–1.631) ***	0.000
Residence		
Urban	1(ref)	
Rural	0.730(0.671–0.794) ***	0.000
Marital status		
Married or cohabitated	1(ref)	
Divorced or separated	1.536(1.116–2.112) **	0.008
Widowed or never married	1.255(1.113–1.415) ***	0.000
Educational level		
No formal schooling	1(ref)	
Less than primary school	0.938(0.832–1.058)	0.298
Primary school completed	0.939(0.835–1.057)	0.297
Secondary/High/Vocational school completed	0.963(0.854–1.087)	0.544
College completed or above	1.325(0.930–1.889)	0.120
Smoking status		
Never	1(ref)	
Former	1.249(1.081–1.444) **	0.003
Current	1.029(0.918–1.155)	0.621
Drinking status		
Never	1(ref)	
Former	1.078(0.935–1.244)	0.301
Current	1.008(0.910–1.117)	0.878
Physical activities		
No	1(ref)	
Yes	0.962(0.887–1.043)	0.349
Participating in social activities		
No	1(ref)	
Yes	1.021(0.942–1.106)	0.615
Hypertension		
No	1(ref)	
Yes	1.479(1.359–1.609) ***	0.000
Kidney disease		
No	1(ref)	
Yes	1.362(1.171–1.583) ***	0.000
Hyperuricemia		
No	1(ref)	
Yes	1.261(1.083–1.469) **	0.003
Fall (s) (last two years)		
No	1(ref)	
Yes	1.021(0.920–1.134)	0.693
Depression		
No	1(ref)	
Yes	1.239(1.135–1.351) ***	0.000
Sleep duration (h)		
≤6	1(ref)	
>6	0.998(0.921–1.081)	0.957
Region of China		
East	1(ref)	
Middle	1.059(0.954–1.175)	0.286
West	1.078(0.972–1.196)	0.156
Northeast	1.505(1.297–1.747) ***	0.000

Notes: CI, confidence interval; * *p* < 0.05; ** *p* < 0.01; *** *p* < 0.001. Model was adjusted for sex, age group, residence, marital status, education, smoking, drinking, physical activities, social activities, hypertension, kidney disease, hyperuricemia, falling, depression, sleep duration, and region.

**Table 3:** Associations and interactions among baseline VAI and HGS with CMM, 2011–2020.

VAI Categories and HGS Categories	HR (95%CI)	*p*-Value	*p*-Interaction
Neither High VAI nor Low HGS	1(Ref)		0.041
High VAI only	1.234(1.119–1.362) ***	0.000	
Low HGS only	1.376(1.213–1.561) ***	0.000	
Both High VAI and Low HGS	1.377(1.120–1.694) **	0.002	

Notes: ** *p* < 0.01; *** *p* < 0.001; Model was adjusted for sex, age group, residence, marital status, education, smoking, drinking, physical activities, social activities, hypertension, kidney disease, hyperuricemia, falling, depression, sleep duration, and region.

## Data Availability

The data that support the findings of this study are openly available in the CHARLS at http://charls.pku.edu.cn (accessed on 26 September 2023). Further inquiries can be directed to the corresponding author.

## Supplementary Materials

The following supporting information can be downloaded at: https://www.mdpi.com/article/10.3390/nu16142277/s1, Figure S1. The nonlinear relationship between VAI and the risk of CMM in all participants. Figure S2. The nonlinear relationship between HGS and the risk of CMM in all participants. Table S1. Relationship of baseline VAI, HGS, and incidence of CMM, 2011–2020. Table S2. Relationship of baseline VAI, HGS, and incidence of CMM, 2011–2020: subgroup analyses. Table S3. Relationship of baseline VAI categories, HGS categories, and incidence of CMM, 2011–2020: subgroup analyses.
