# Supplementary material for: Association of Visceral Adiposity Index and Handgrip Strength with Cardiometabolic Multimorbidity among Middle-Aged and Older Adults: Findings from Charls 2011–2020
Source: Nutrients. 2024 Jul 15;16(14):2277. doi: 10.3390/nu16142277 (PMC11280108; doi:10.3390/nu16142277)
Supplement: Supplementary file 1 [file nutrients-16-02277-s001.zip › nutrients-3085216-supplementary.pdf]

Supplementary Material

Figure. S1. The non-linear relationship between VAI and the risk of CMM in all participants.

Figure. S2. The non-linear relationship between HGS and the risk of CMM in all participants.

Table S1. Relationship of baseline VAI, HGS, and incidence of CMM, 2011-2020.

Table S2. Relationship of baseline VAI, HGS, and incidence of CMM, 2011-2020: subgroup analyses.

Table S3. Relationship of baseline VAI categories, HGS categories, and incidence of CMM, 2011-2020: subgroup analyses.

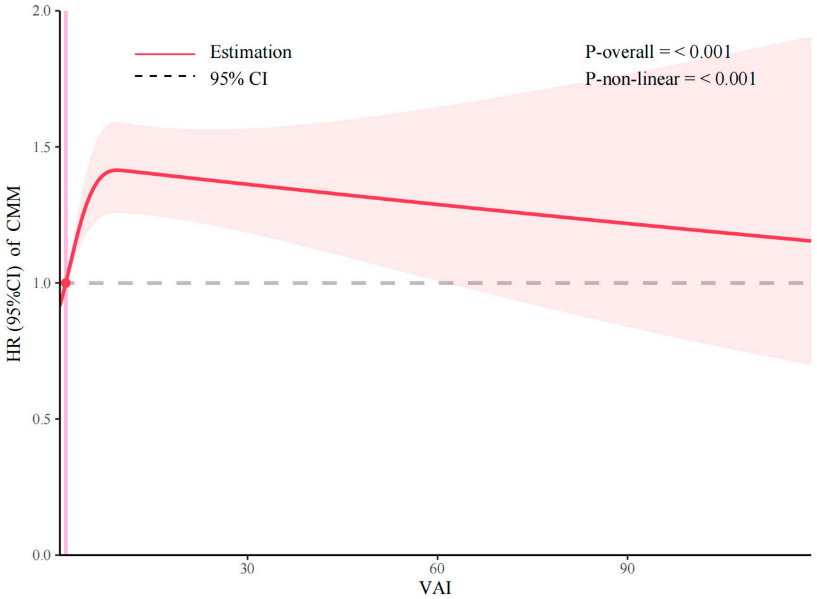

Figure. S1 The non-linear relationship between VAI and the risk of CMM in all participants. A non-linear relationship was detected after adjusting for sex, age group, residence, marital status, education, smoking, drinking, physical activities, social activities, hypertension, kidney disease, hyperuricemia, fall, depression, sleep duration, region.

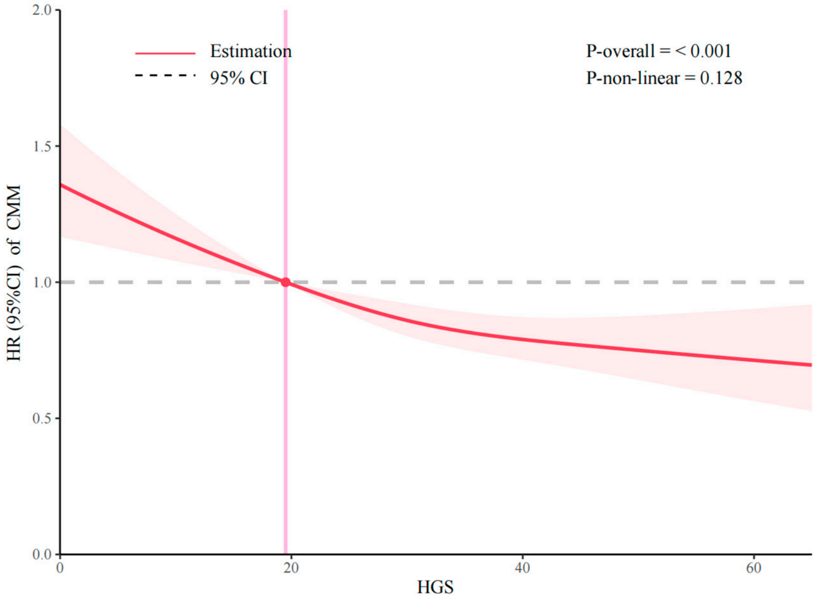

Figure. S2 The non-linear relationship between HGS and the risk of CMM in all participants. A non-linear relationship was detected after adjusting for sex, age group, residence, marital status, education, smoking, drinking, physical activities, social activities, hypertension, kidney disease, hyperuricemia, fall, depression, sleep duration, region.

Table. S1. Relationship of baseline VAI, HGS, and incidence of CMM, 2011-2020.

Variables	Individuals	Events	HR (95%CI)	*P*-value
VAI score	7909	2501	1.003 (1.000-1.006)	0.092
HGS (kg)			0.990 (0.985-0.994) ***	0.000

*$p < 0.05$; **$p < 0.01$; ***$p <0.001$

Model was adjusted for sex, age group, residence, marital status, education, smoking, drinking, physical activities, social activities, hypertension, kidney disease, hyperuricemia, fall, depression, sleep duration, region.

†VAI, HGS is analyzed as a continuous variable in the table.

Table S2 Relationship of baseline VAI, HGS, and incidence of CMM, 2011-2020: subgroup analyses.

Variables		Individuals	Events	HR (*95%CI*)	*P*-value
Sex	Male				
	VAI score	3704	1254	1.002 (0.996-1.007)	0.560
	HGS (kg)			0.990 (0.983-0.996) **	0.001
	Female				
	VAI score	4205	1247	1.004 (1.000-1.009)	0.057
	HGS (kg)			0.991 (0.983-0.999) *	0.026
Age group	45-64				
	VAI score	5820	1576	1.004 (0.999-1.008)	0.086
	HGS (kg)			0.995 (0.988-1.001)	0.079
	≥65				
	VAI score	2089	925	1.003 (0.996-1.011)	0.415
	HGS (kg)			0.982 (0.974-0.990) ***	0.000
Region of China	East				
	VAI score	2344	682	1.002 (0.993-1.010)	0.711
	HGS (kg)			0.993 (0.984-1.003)	0.178
	Middle				
	VAI score	2426	767	1.004 (0.995-1.012)	0.376
	HGS (kg)			0.985 (0.977-0.994) **	0.001
	West				
	VAI score	2570	811	1.002 (0.997-1.007)	0.343
	HGS (kg)			0.989 (0.981-0.997) *	0.010
	Northeast				
	VAI score	569	241	1.010 (0.998-1.022)	0.112
	HGS (kg)			0.990 (0.974-1.007)	0.238

*$p < 0.05$; **$p < 0.01$; ***$p < 0.001$

Model was adjusted for sex (unadjusted in subgroup analysis stratified by sex), age group (unadjusted in subgroup analysis stratified by age), residence, marital status, education, smoking, drinking, physical activities, social activities, hypertension, kidney disease, hyperuricemia, fall, depression, sleep duration, region (unadjusted in subgroup analysis stratified by region).

†VAI, HGS is analyzed as a continuous variable in the table.

Table S3. Relationship of baseline VAI categories, HGS categories, and incidence of CMM, 2011-2020: subgroup analyses.

Variables		Individuals	Events	HR (*95%CI*)	*P*-value
Sex	Male				
	VAI categories				
	normal	3115	1028	1(ref)	
	high	589	226	1.155 (0.994-1.342)	0.059
	HGS categories				
	normal	3246	1027	1(ref)	
	low	458	227	1.407 (1.207-1.639) ***	0.000
	Female				
	VAI categories				
	normal	2817	756	1(ref)	
	high	1388	491	1.233 (1.097-1.385) ***	0.000
	HGS categories				
	normal	3712	1053	1(ref)	
	low	493	194	1.181 (1.005-1.388) *	0.043
Age group	45-64				
	VAI categories				
	normal	4295	1079	1(ref)	
	high	1525	497	1.219 (1.091-1.363) ***	0.000
	HGS categories				
	normal	5396	1444	1(ref)	
	low	424	132	1.094 (0.912-1.312)	0.334
	≥65				
	VAI categories				
	normal	1637	705	1(ref)	
	high	452	220	1.179 (1.001-1.388) *	0.048
	HGS categories				
	normal	1562	636	1(ref)	
	low	527	289	1.449 (1.256-1.671) ***	0.000
Region of China	East				
	VAI categories				
	normal	1757	473	1(ref)	
	high	587	209	1.238 (1.042-1.470) *	0.015
	HGS categories				
	normal	2111	590	1(ref)	
	low	233	92	1.211 (0.958-1.531)	0.109
	Middle				

VAI categories				
normal	1769	543	1(ref)	
high	657	224	1.043 (0.886-1.227)	0.615
HGS categories				
normal	2107	632	1(ref)	
low	319	135	1.308 (1.076-1.591) **	0.007
West				
VAI categories				
normal	2001	602	1(ref)	
high	569	209	1.303 (1.101-1.542) **	0.002
HGS categories				
normal	2238	654	1(ref)	
low	332	157	1.402 (1.166-1.687) ***	0.000
Northeast				
VAI categories				
normal	405	166	1(ref)	
high	164	75	1.244 (0.926-1.671)	0.148
HGS categories				
normal	502	204	1(ref)	
low	67	37	1.119 (0.766-1.634)	0.562

*p < 0.05; **p < 0.01; ***p <0.001

Model was adjusted for sex (unadjusted in subgroup analysis stratified by sex), age group (unadjusted in subgroup analysis stratified by age), residence, marital status, education, smoking, drinking, physical activities, social activities, hypertension, kidney disease, hyperuricemia, fall, depression, sleep duration, region (unadjusted in subgroup analysis stratified by region).

†VAI, HGS is analyzed as a categorical variable in the table.
